# Development of the Intimate Partner Violence During Pregnancy Instrument (IPVPI)

**DOI:** 10.3389/fpubh.2019.00043

**Published:** 2019-03-21

**Authors:** Satomi Doi, Takeo Fujiwara, Aya Isumi

**Affiliations:** Department of Global Health Promotion, Tokyo Medical and Dental University, Tokyo, Japan

**Keywords:** intimate partner violence, physical abuse, verbal abuse, pregnant women, risk factors, Japan

## Abstract

**Background:** Intimate partner violence (IPV) during pregnancy can lead to negative consequences for both the mother and offspring. Although IPV is recognized as a worldwide public health issue, its prevalence is considered to be underestimated because cases are likely underreported, suggesting that there might be unmeasured IPV. The aim of this study was to develop an instrument to detect IPV in pregnant women.

**Methods:** A total of 6,590 women in Aichi prefecture, Japan, who took part in a 3 or 4 month infant health checkup program, participated in the study. Questionnaires assessing history of IPV during pregnancy (physical abuse and verbal abuse), maternal characteristics, partner's characteristics, and household characteristics were mailed to women before, or distributed at, the checkup. Women returned the questionnaires to the checkup sites or mailed them back to the health centers. A prediction model for history of IPV was then generated using potential risk factors selected based on the literature.

**Results:** Among 6,530 women who responded to either question on IPV during pregnancy (response rate = 67.3%), the rate of participants who experienced any IPV during pregnancy was 11.1% (physical IPV = 1.2%; verbal IPV = 10.8%). Multiple logistic regression analyses showed that maternal age (<25 years old), multiparity, history of artificial abortion, negative feelings when the pregnancy was confirmed (e.g., confused), having no one to provide support during pregnancy, having relationship problems with their partner, paternal smoking during pregnancy, and difficult financial status were associated with any abuse from the partner. Based on the analysis, the Intimate Partner Violence during Pregnancy Instrument (IPVPI) was developed, comprising of eight questions to detect unmeasured IPV in pregnant women, and showed moderate predictive power (area under receiver operating characteristic curve = 0.719, 95% confidence interval: 0.698 to 0.740) ranging from 0 to 16 with a cut-off point of 2 (sensitivity = 79.5%, specificity = 47.1%).

**Conclusion:** The IPVPI, which allows to ask indirect questions rather that asking directly about experience of IPV, might be helpful to detect unmeasured IPV in pregnant women in fields of primary healthcare and obstetrics. Further research longitudinal studies are needed to improve the sensitivity and specificity of the IPVPI.

## Introduction

Intimate partner violence (IPV), refers to “one of the most common forms of violence against women and includes physical, sexual, and emotional abuse and controlling behaviors by an intimate partner” (1, 2). IPV is recognized as a worldwide public health issue (3), and types of IPV include physical violence, sexual violence, emotional abuse, and controlling behaviors such as economic abuse (1). Lifetime prevalence of physical or sexual IPV among ever-partnered women has been estimated at 29.8% (95% CI = 25.8 to 33.9) in the United States (US), 25.4% (95% CI = 20.9 to 30.0) in Europe, and 23.2% (95% CI = 20.1 to 29.0) in high-income regions including Japan (3). The cost of IPV as well as women's physical and mental health is notable. In the US, the annual costs of IPV, especially intimate partner rape, physical assault, and stalking, were estimated to be more than $US5.8 billion (4). Of this total amount, direct costs of medical and mental healthcare services amounted to approximately $US4.1 billion, costs of lost productivity from paid work and household work among victims of non-fatal IPV came to $US0.9 billion, while $US0.9 billion in lifetime wages were lost among victims of IPV homicide (4).

IPV during pregnancy can lead to negative consequences for both the mother and offspring, including maternal suicide (5), maternal alcohol abuse and smoking (6), delayed prenatal care (6, 7), low birth weight (8, 9), miscarriage (10), and postpartum depression (11). Thus, there is a need to prevent the harmful effects of IPV in pregnancy. Although Japan's low prevalence of physical IPV during pregnancy is estimated at 1% (12), the prevalence of IPV in Japan is increasing (13). Moreover, its prevalence is considered to be underestimated because cases are likely underreported; previous studies have indicated that Japanese women are less likely to report IPV due to feelings of shame (14, 15). Yahata (16) suggested that IPV remains hidden in an abused woman's family until she dies. To reveal the current situation, the development of a screening tool is needed to estimate the possibility of IPV. Fortunately in Japan, all pregnant women must submit a pregnancy notification form to their local government office during early pregnancy, and have access to postpartum examinations. Using this unique system, municipal governments have the opportunity to assist for pregnant women experience IPV during pregnancy and after delivery. Therefore, it is possible to detect unmeasured IPV in those pregnant women by using information collected from the local government's pregnancy notification forms.

To develop a screening tool for unmeasured IPV in pregnant women, several risk factors for IPV during pregnancy need to be reviewed. In a meta-analysis of 55 articles about pregnant women in developed countries, James et al. (17) showed that lower socioeconomic status, living together with others, and unintended pregnancy can significantly predict physical abuse during pregnancy. Additionally, Finnbogadóttir et al. (18), who conducted a population-based survey of pregnant women in Sweden, showed that maternal depressive symptoms were associated with physical, sexual, and emotional abuse during pregnancy. The main risk factors for IPV regardless of pregnancy have been identified as young maternal and young partner age (19–21), unemployment among women and their partners (21–23), low household income (19, 21), relationship problems with their partner (23), and partner's alcohol abuse (20–24). Although not statistically significant, maternal consumption of alcohol (22, 23), maternal and paternal smoking (23), multiparity, and history of abortion (18) were found to be possible risk factors.

In Japan, a few previous studies found that multiparity (25–27), previous physical violence from a partner (25, 26), old maternal and young partner age (27), previous abortion experience (27) are possible risk factors among pregnant women and women in perinatal setting. However, the risk factors for IPV during pregnancy are still unclear because Japanese previous studies were not a population-based study and had small sample size. Although the risk factors for IPV during pregnancy are not completely consistent according to country, race, culture, and values (18), there is a need to identify the risk factors for IPV during pregnancy in Japan, which may also apply to other countries.

The aim of this study was to develop an instrument—the Intimate Partner Violence during Pregnancy Instrument (IPVPI)—that can detect unmeasured IPV in pregnant women during pregnancy, and which can be incorporated in local governments' pregnancy notification forms.

## Methods

### Participants and Procedures

Details of the study protocol have been published elsewhere (11). We invited all 54 municipalities in Aichi, Japan, to participate in this study. Nagoya city, the prefectural capital of Aichi, and 45 municipalities agreed to participate. The study targeted women who registered to take part in a 3 or 4 month infant health checkup program conducted by municipal governments at public health centers from October to November 2012 (*N* = 9,707). Almost all women participated in the checkup program (participation rate = 97.9%). Of 9,707 women, 6,590 responded to the mailed anonymous questionnaire, which assessed women's exposure to IPV during pregnancy and other possible risk factors. For 34 municipalities, the anonymous questionnaire was mailed to women before the health checkup program and was collected at the health checkup sites (response rate = 77%). For 11 municipalities, the anonymous questionnaire was given to women at the health checkup sites and was later mailed back to the health centers (response rate = 48%).

### Measurements

#### IPV During Pregnancy

Participants were asked the following two questions about IPV during pregnancy in Japanese: “Have you been slapped or beaten up by your partner during pregnancy while having a fight?” (physical IPV) and “Have you been verbally humiliated or yelled at by your partner during pregnancy?” (verbal IPV). Response questions were “never,” “a few times,” “sometimes,” and “often.” These questions were developed based on the revised Conflict Tactics Scale (28) and were used in a previous study (11). To minimize the burden on participants, only two questions were selected and used in this study.

#### Possible Risk Factors

The questionnaire assessed the following factors: participants' maternal age; parity (“primipara” or “multipara”); history of natural abortion, preterm, stillbirth, and artificial abortion; the woman's feelings when her pregnancy was confirmed (“happy,” “unexpected but happy,” “unexpected and confused,” “did not know what to do,” or “no feelings”), having someone to provide support during pregnancy (“yes” or “no”), having relationship problems with their partner during pregnancy, history of smoking during pregnancy (“yes,” “stopped after pregnancy was confirmed,” or “no”), history of drinking alcohol during pregnancy (“yes” or “no”), and employment status (“full-time,” “part-time,” or “not working”). Partners' demographics, including age, history of smoking during pregnancy (“yes” or “no”), and employment status (“full-time,” “part-time,” or “not working”) were filled out by the participants. Further, the household characteristics of living together with others and financial status (“stable,” “able to manage,” “difficult to manage,” or “unstable”) were also assessed.

### Statistical Analysis

Of the women who gave a response to the questionnaire (*N* = 6,590), 6,530 women who responded to either question on IPV during pregnancy were used for the analysis. Those who did not respond to the questions on IPV during pregnancy significantly smoked more cigarettes (*p* = 0.007) than those who did respond to the questions.

To explore risk factors for IPV during pregnancy, multiple logistic regression analysis was conducted. In the analysis, the outcome variables were IPV variables (either physical or verbal IPV)—participants who responded “never” to both physical and verbal abuse questions were categorized as having no experience of IPV, and participants who responded “a few times,” “sometimes,” and “often” for either question on physical or verbal abuse were categorized as having experience of IPV. First, simple logistic regression was performed to determine the crude association of each risk factor on IPV. In addition to the crude model, multiple logistic regression analysis included all risk factor variables that were fitted in the model. To develop the IPVPI, risk factors that showed a significant association with IPV during pregnancy in the multiple logistic regression analysis were selected. To create formula that can predict IPV using the selected risk factors, odds ratios (OR) from the multiple logistic regression analysis were used for weighting the risk factors. The weighting system based on a previous study (29) was carried out as follows: the score was not weighted when ORs ranged from 1.00 to 1.49; the score was doubled when ORs ranged from 1.50 to 2.49; the score was tripled when ORs ranged from 2.50 to 3.49, and the score was 6 times when ORs ranged 5.50 to 6.49. However, the cut-off for the ORs may be flexible to increase the area under the curve (AUC). Data were analyzed using STATA version 14.1.

## Results

### Prevalence of Any IPV During Pregnancy and Demographic Data

Table 1 shows the prevalence of IPV during pregnancy, including missing data. Of the participants, 1.2% experienced physical IPV and 10.8% experienced verbal IPV. The total number of participants who experienced any IPV during pregnancy (physical and/or verbal IPV) was 730 (11.1%).

**Table 1:** Prevalence of physical and verbal IPV during pregnancy.

	**Participants (*****N*** **= 6,590)**	
	***N***	**%**
**PHYSICAL IPV DURING PREGNANCY**		
Never	6,448	97.9
A few times	53	0.8
Sometimes	19	0.3
Often	6	0.1
Missing	64	1.0
**VERBAL IPV DURING PREGNANCY**		
Never	5,795	87.9
A few times	437	6.6
Sometimes	243	3.7
Often	35	0.5
Missing	80	1.2
**ANY IPV DURING PREGNANCY**^**a**^		
Never	5,800	88.0
A few times/sometimes/often	730	11.1
Missing^b^	60	0.9

a *“Any IPV during pregnancy” means the experiences of physical and/or verbal abuse*.

b Participants who did not answer both physical and verbal abuse questions were classified as “missing.”

In addition, demographic data of participants who experienced any IPV during pregnancy or no IPV are shown in Table 2. Participants who experienced IPV during pregnancy were younger; had a younger partner; had a higher rate of artificial abortion; were less happy when their pregnancy was confirmed; had fewer people to provide support during pregnancy; were more likely to have relationship problems with their partner, smoke cigarettes, drink alcohol, and to have a partner who smoked; and were less likely to work full-time and have a partner who worked full-time than those who did not experience any IPV during pregnancy.

**Table 2:** Demographic data of all participants.

		**Any abuse from partner**	
		**Yes (*N* = 730)**	**No (*N* = 5,800)**
Maternal age^**^	≥25	612 (83.8)	5,388 (92.9)
	<25	117 (16.0)	398 (6.9)
	Missing	14 (0.2)	1 (0.1)
Paternal age^**^	≥25	638 (87.4)	5,520 (95.2)
	<25	76 (10.4)	231 (4.0)
	Missing	16 (2.2)	49 (0.8)
Living together with others^*^	No	692 (94.8)	5,604 (96.6)
	Yes, living with others	38 (5.2)	196 (3.4)
Parity	Primipara	341 (46.7)	2,949 (50.8)
	Multipara	388 (53.2)	2,834 (48.9)
	Missing	1 (0.1)	17 (0.3)
History of natural abortion	None	608 (83.3)	4,761 (82.1)
	≥ 1	122 (16.7)	1,039 (17.9)
History of preterm	None	718 (98.4)	5,722 (98.7)
	≥ 1	12 (1.6)	78 (1.3)
History of stillbirth	None	723 (99.0)	5,736 (98.9)
	≥ 1	7 (1.0)	64 (1.1)
History of artificial abortion^**^	None	639 (87.5)	5,439 (93.8)
	≥ 1	91 (12.5)	361 (6.2)
Feelings when pregnancy was confirmed^**^	Happy	439 (60.1)	4,475 (77.2)
	Unexpected but happy	176 (24.1)	936 (16.1)
	Unexpected and confused/Did not know what to do/No feelings/Other	113 (15.5)	374 (6.4)
	No response	2 (0.3)	15 (0.3)
Having someone to provide support during pregnancy^**^	Yes	692 (94.8)	5,457 (97.5)
	No	35 (4.8)	127 (2.2)
	Missing	3 (0.4)	16 (0.3)
Having relationship problems with partner^**^	Yes	179 (24.5)	216 (3.7)
	No	546 (74.8)	5,553 (95.7)
	Missing	5 (0.7)	31 (0.5)
Maternal history of smoking during pregnancy ^**^	Yes/stopped after pregnancy was confirmed	149 (20.4)	558 (9.6)
	No	581 (79.6)	5,239 (90.3)
	Missing	0 (0.0)	3 (0.1)
Paternal smoking during pregnancy^**^	Yes	183 (25.1)	785 (13.5)
	No	546 (74.8)	5,003 (86.3)
	Missing	1 (0.1)	12 (0.2)
Maternal history of drinking alcohol during pregnancy^**^	Yes	42 (5.8)	215 (3.7)
	No	685 (93.8)	5,577 (96.2)
	Missing	3 (0.4)	8 (0.1)
Financial status^**^	Stable	200 (27.4)	2,674 (46.1)
	Able to manage	321 (43.9)	2,328 (40.1)
	Difficult to manage or unstable	170 (23.3)	564 (9.7)
	Missing	39 (5.3)	234 (4.0)
Maternal employment status^*^	Full-time	110 (15.1)	962 (16.6)
	Part-time	50 (6.9)	264 (4.6)
	Not working	556 (76.2)	4,496 (77.5)
	Missing	14 (1.9)	78 (1.3)
Paternal employment status^**^	Full-time	677 (92.7)	5,616 (96.8)
	Part-time	13 (1.8)	43 (0.7)
	Not working	10 (1.4)	46 (0.8)
	Missing	30 (4.1)	95 (1.6)

* *p < 0.05 (χ^2^ test)*.

** *p < 0.01 (χ^2^ test)*.

### Risk Factors for IPV During Pregnancy

Table 3 shows the results of crude and multiple logistic regression analyses. Risk factors that showed a significant association with IPV in the adjusted model were maternal age <25 years old (OR = 1.41, 95% confidence interval (CI) = 1.03 to 1.92), multipara (OR = 1.38, 95% CI = 1.16 to 1.64), history of artificial abortion (OR = 1.44, 95% CI = 1.10 to 1.90), feeling unexpected but happy when pregnancy was confirmed (OR = 1.41, 95% CI = 1.15 to 1.73), feeling unexpected and unhappy (unexpected and confused, did not know what to do, no feelings, other) when pregnancy was confirmed (OR = 1.85, 95% CI = 1.43 to 2.39), having no one to provide support during pregnancy (OR = 1.66, 95% CI = 1.09 to 2.53), having relationship problems with partner during pregnancy (OR = 6.39, 95% CI = 5.05 to 8.08), paternal smoking during pregnancy (OR = 1.47, 95% CI = 1.19 to 1.82), manageable financial status (OR = 1.51, 95% CI = 1.25 to 1.84), difficult or unstable financial status (OR = 2.40, 95% CI = 1.87 to 3.09), and no response to the question of financial status (OR = 1.63, 95% CI = 1.06 to 2.51).

**Table 3:** Odds ratios and 95% confidence intervals for any IPV during pregnancy by maternal, partner, or household demographics.

		**Any IPV during pregnancy**			
		**Crude**		**Multiple**	
		**OR**	**95% CI**	**OR**	**95% CI**
Maternal age	≥25	Reference		Reference	
	<25	**2.59**	2.07–3.23	**1.41**	1.03–1.92
	No response	0.63	0.08–4.79	0.35	0.04–3.50
Paternal age	≥25	Reference		Reference	
	<25	**2.85**	2.17–3.74	1.41	0.97–2.04
	No response	**2.83**	1.60–5.00	1.35	0.52–3.49
Living together with others	No	Reference		Reference	
	Yes, living with others	**1.57**	1.10–2.24	1.15	0.77–1.71
Parity	Primiparity	Reference		Reference	
	Multipara	**1.18**	1.01–1.38	**1.38**	1.16–1.64
	No response	0.51	0.07–3.83	0.28	0.03–2.56
History of natural abortion	None	Reference		Reference	
	≥1	0.92	0.75–1.13	0.91	0.72–1.13
History of preterm	None	Reference		Reference	
	≥1	2.92	0.90–9.45	1.30	0.68–2.46
History of stillbirth	None	Reference		Reference	
	≥1	0.87	0.40–1.90	0.61	0.26–1.44
History of artificial abortion	None	Reference		Reference	
	≥1	**2.15**	1.68–2.74	**1.44**	1.10–1.90
Feelings when pregnancy was confirmed	Happy	Reference		Reference	
	Unexpected but happy	**1.92**	1.59–2.31	**1.41**	1.15–1.73
	Unexpected and confused/Did not know what to do/ No feelings/Other	**3.08**	2.44–3.88	**1.85**	1.43–2.39
	No response	1.36	0.31–5.96	0.90	0.19–4.31
Having someone to provide support during pregnancy	Yes	Reference		Reference	
	No	**2.25**	1.54–3.30	**1.66**	1.09–2.53
	No response	1.53	0.45–5.27	1.64	0.45–5.96
Having relationship problems with partner	No	Reference		Reference	
	Yes	**8.43**	6.79–10.47	**6.39**	5.05–8.08
	No response	1.64	0.64–4.24	1.48	0.56–3.91
Maternal history of smoking during pregnancy	No	Reference		Reference	
	Yes^a^	**2.41**	1.97–2.94	1.22	0.96–1.56
	No response	NA		NA	
Paternal smoking during pregnancy	No	Reference		Reference	
	Yes	**2.14**	1.78–2.57	**1.47**	1.19–1.82
	No response	0.76	0.10–5.88	0.42	0.05–3.72
Maternal history of drinking alcohol during pregnancy	No	Reference		Reference	
	Yes	**1.59**	1.13–2.23	1.29	0.89–1.86
	No response	3.05	0.81–11.54	3.46	0.79–15.09
Financial status	Stable	Reference		Reference	
	Able to manage	**1.84**	1.53–2.22	**1.51**	1.25–1.84
	Difficult to manage/unstable	**4.03**	3.22–5.04	**2.40**	1.87–3.09
	No response	**2.23**	1.54–3.22	**1.63**	1.06–2.51
Maternal employment status	Full-time	Reference		Reference	
	Part-time	**1.66**	1.15–2.38	0.98	0.66–1.45
	Not working	1.08	0.87–1.34	0.79	0.62–0.99
	No response	1.57	0.86–2.87	1.02	0.42–2.46
Paternal employment status	Full-time	Reference		Reference	
	Part-time	**2.51**	1.34–4.69	1.23	0.61–2.49
	Not working	1.80	0.91–3.59	0.65	0.29–1.45
	No response	**2.62**	1.72–3.98	1.16	0.52–2.57

a *“Yes” also includes stopping after pregnancy was confirmed. The bold values mean significant*.

### Development of the IPVPI

Based on the multiple logistic analysis, we created the following formula to predict IPV during pregnancy:

Ln ($\frac{p}{\left(1-p\right)}$) = 0.053 + maternal age less than 25 years old + multipara + history of artificial abortion + feeling unexpected but happy when pregnancy was confirmed + 2^*^
feeling unexpected and unhappy + 2^*^
having no one to provide support during pregnancy + 6^*^
having relationship problems with partner + paternal smoking during pregnancy +
manageable financial status + 2^*^ difficult or unstable financial status + 2^*^
no response to the question of financial status

where *p* denotes probability of having IPV, either physical or verbal, during pregnancy. As for financial status, the score of “able to manage” was not weighted as double, although the OR was 1.51 (95% CI = 1.25 to 1.84), in order to distinguish “difficult to manage or unstable,” and this weighting showed better AUC.

Using this formula, we calculated AUC, sensitivity, specificity, and the overall rate of correct classification using the total score of the IPVPI. The AUC of the IPVPI total score was 0.719 (95% CI = 0.698 to 0.740), which indicates moderate accuracy of the scale (30, 31). According to sensitivity, specificity, and the overall rate of correct classification, the cut-off point of the IPVPI was 2, in which sensitivity, specificity, and the overall rate of correct classification were 79.5, 47.2, and 50.8%, respectively (Table 4). From these results, we developed the IPVPI (Appendix in Supplementary Material).

**Table 4:** Prediction parameters for the IPVPI total score.

**Score**	**Sensitivity**	**Specificity**	**Overall rate of correct classification**	**LR+**	**LR–**
0	100.0	0.0	11.2	1.00	
1	93.8	16.7	25.3	1.13	0.37
2	79.5	47.2	50.8	1.51	0.44
3	61.1	71.9	70.7	2.18	0.54
4	45.8	86.5	82.0	3.40	0.63
5	34.8	92.9	86.4	4.90	0.70
6	27.7	95.2	87.7	5.79	0.76
7	24.5	96.3	88.3	6.63	0.79
8	20.4	97.4	88.8	7.84	0.82
9	15.5	98.3	89.0	8.98	0.86
10	9.6	99.1	89.1	10.91	0.91
11	5.1	99.6	89.0	13.36	0.95
12	1.9	99.9	88.8	18.54	0.98
13	0.4	99.9	88.8	23.83	0.99
14+	0.0	100.0	88.8		1.00

*IPVPI, Intimate partner violence during pregnancy instrument; LR+, Positive likelihood ratio; LR–, Negative likelihood ratio*.

## Discussion

This is the first study to develop a screening scale to detect unmeasured IPV in pregnant women. The IPVPS is comprised of eight questions: maternal age, multiparity, history of artificial abortion, feelings when pregnancy was confirmed, someone to provide support during pregnancy, relationship with partner, paternal smoking during pregnancy, and financial status. The novelty of the IPVPI is that this scale can detect the existence of IPV risk without asking specific questions about IPV, which is useful because pregnant women hesitate to respond existence of IPV when asked specifically. The total score of the IPVPI, which was weighted for each question, showed moderate accuracy (AUC = 0.719, 95% CI: 0.698 to 0.740).

The IPVPI may be useful to detect unmeasured IPV during pregnancy by the primary healthcare provider in maternal and child health settings. Public health nurses or midwives at hospital obstetrics or gynecology departments can use the scale in the early stages of pregnancy to identify unmeasured IPV in pregnant women by assessing women against the IPVPI at the time of pregnancy notification. For pregnant women who showed higher IPVPI scores, even if they did not mention the existence of IPV, public health nurses may assist them by seeking a more detailed assessment of IPV or providing adequate support to protect them from IPV. Detection of unmeasured IPV in pregnant women and providing adequate support seems important because IPV during pregnancy can lead to maternal suicide (5), delayed prenatal care (6, 7), maternal alcohol abuse and smoking (6), low birth weight (8, 9), miscarriage (10), and postpartum depression (11). Further studies showing the effectiveness of preventing these adverse outcomes using the IPVPI are warranted.

A previous systematic review (32) found that the several screening scales for IPV were valid and reliable: the Abuse Assessment Screen (33), Partner Violence Screen (34), and Violence Against Women Screen (35). However, these previous screening scales assessed the existence of IPV by posing questions focused on actual IPV. Thus, the IPVPI is unique in identifying IPV during pregnancy without asking about actual IPV. In other words, it includes questions that are only related to pregnant women's and partners' demographics. Therefore, the IPVPI might be useful for detecting those pregnant women who are reluctant to disclose IPV. To the best of our knowledge, no other scales have been developed that detect unmeasured IPV in pregnant women without asking about the existence of IPV.

The IPVPI is composed of eight questions that cover the risk factors for IPV found in previous studies. Other possible risk factors identified from the literature were not found to be associated with IPV during pregnancy in our multiple logistic regression analyses, such as paternal age (18, 20, 21, 23); living with others (23); history of preterm, stillbirth, and natural abortion (18); maternal history of smoking during pregnancy (23), maternal history of drinking alcohol during pregnancy (22, 23), maternal employment status (18, 21, 23), and paternal employment status (21–23). However, almost all of these variables were significantly associated with IPV during pregnancy in our crude model. In terms of paternal age, Kyriacou et al. (22), who conducted a multi-country survey that included Japan, also reported that no association of paternal age with IPV was found in the multivariate models due to multicollinearity with maternal age. Living with others may also face the problem of collinearity with maternal age: according to government figures from 2009, about 80% of Japanese mothers aged 15–19 years and about 60% of mothers aged 20–23 years experienced premarital pregnancy, whereas only 20% of mothers aged over 25 years experienced premarital pregnancy (36).

We assessed physical IPV during pregnancy as “Have you been slapped or beaten up by your partner during pregnancy while having a fight?” This does not reflect Japanese culture that Japanese male partner may slap without fighting. This is simply to capture physical IPV, which occur during fighting, which was also used in Conflict Tactics Scale (28). Further, slapping without fighting should be physical IPV, but the case must be rare.

As for maternal history of smoking and drinking alcohol during pregnancy, these factors were confounded with socioeconomic status represented as financial status in this study. Previous studies showed that higher socioeconomic status is associated with drinking alcohol during pregnancy (37, 38) and lower socioeconomic status is associated with smoking (39) and also smoking during pregnancy (40). Similarly, maternal and paternal employment status can be associated with financial status (41), which might not be commonly assessed in a public health setting. Therefore, the possible risk factors for IPV during pregnancy may be different in other countries. Further studies are needed to confirm the current questions used in the IPVPI and to explore cultural differences in possible risk factors for IPV during pregnancy.

This study has several limitations. First, women with a higher risk of IPV may be less involved in this study, suggesting selection bias. That is, association between risk factors and IPV might be underestimated. Second, IPV might be underreported or overreported due to information bias, which warrant further study using richer data. Nonetheless, we conducted study using the anonymous questionnaire to avoid information bias as much as possible. Third, we assessed only physical and verbal IPV during pregnancy. Other types of IPV, such as sexual violence and controlling behaviors (42), were not evaluated. Nonetheless, we developed a scale to detect the possibility of physical and psychological IPV based on information obtained from the pregnancy notification form. Fourth, the sensitivity and specificity of the IPVPI was not high. Because IPVPI is screening tool to detect IPV, further research longitudinal studies using real data are needed to improve IPVPI which evaluates IPV and maternal characteristics during pregnancy. Fifth, this study did not examine the other important risk factors for IPV, such as adverse childhood experiences (17, 19, 21, 24). Thus, the accuracy of the IPVPI might improve by adding this and other risk factors to the scale. However, as the response rate to questions on adverse childhood experiences might be low (19), this point should be carefully considered. Sixth, the participants of the current study were Japanese women, thus generalizability of IPVPI to other population is limited.

In conclusion, the eight-item IPVPI, which does not directly ask about the existence of IPV, might be helpful in the fields of primary healthcare and obstetrics to identify unmeasured IPV in pregnant women. However, it is also noted that the accuracy of the IPVPI was under ideal conditions. Further studies confirming the usability of the IPVPI in several cultural settings and improving the accuracy of the instrument are needed.

## Ethics Statement

This study was approved by the Ethics Committee of the National Center for Child Health and Development (reference number 611).

## Author Contributions

TF designed the study and managed the administration of the study, including the ethical review process. SD analyzed the data and drafted the manuscript. TF and AI provided critical comments on the manuscript, related to intellectual content. All authors have read and approved the final manuscript.

### Conflict of Interest Statement

The authors declare that the research was conducted in the absence of any commercial or financial relationships that could be construed as a potential conflict of interest.
