# Supplementary material for: Development of the Intimate Partner Violence During Pregnancy Instrument (IPVPI)
Source: Front Public Health. 2019 Mar 21;7:43. doi: 10.3389/fpubh.2019.00043 (PMC6437061; doi:10.3389/fpubh.2019.00043)
Supplement: Supplementary file 1 [file Data_Sheet_1.docx]

Appendix

| Question | | Answer | Score |
| --- | --- | --- | --- |
| 1 | How old are you? | □ Under 25 years old | 1 |
|  |  | □ 25 years and over | 0 |
| 2 | Is this your first baby? | □ Yes | 0 |
|  |  | □ No | 1 |
| 3 | Have you ever experienced artificial abortion before this pregnancy? | □ No | 0 |
|  |  | □ Yes | 1 |
| 4 | How did you feel when you found out you were pregnant? | □ Happy | 0 |
|  |  | □ Unexpected but happy | 1 |
|  |  | □ Unexpected and confused | 2 |
|  |  | □ Did not know what to do | 2 |
|  |  | □ No feelings | 2 |
|  |  | □ Other | 2 |
| 5 | Do you have someone to support you when you have problems during your pregnancy? | □ Yes | 0 |
|  |  | □ No | 2 |
| 6 | Do you have problems or worries about your partner relationship during pregnancy? | □ No | 0 |
|  |  | □ Yes | 6 |
| 7 | Do your partner or any family members you live with smoke in the same room as you during your pregnancy? | □ No | 0 |
|  |  | □ Yes | 1 |
| 8 | How is the economic situation of your household? | □ Stable | 0 |
|  |  | □ Able to manage | 1 |
|  |  | □ Difficult to manage | 2 |
|  |  | □ Unstable | 2 |
|  |  | □ Do not want to answer | 2 |
| Total score (≥ 2 indicates a high risk for any abuse from partner during pregnancy) | | | ( ) |
